# Simple Python‐based methods for analysis and drift‐correction of STM images

**DOI:** 10.1111/jmi.13426

**Published:** 2025-05-14

**Authors:** Francesco Cazzadori, Alessandro Facchin, Silvio Reginato, Christian Durante

**Affiliations:** ^1^ Department of Chemical Sciences University of Padova Padova Italy; ^2^ Department of Chemistry TUM School of Natural Sciences Garching Germany

**Keywords:** drift, EC‐STM, high throughput, image analysis, porphyrin, Python

## Abstract

A successful scanning tunnelling microscopy (STM) experiment relies on both delicate sample preparation and measurement, and careful image filtering and analysis to provide clear and solid results. Processing and analysis of STM images may result in a tricky task, due to the complexity and specificity of the probed systems. In this paper, we introduce our recently developed, simple Python‐based methods for filtering and analysing STM images, with the aim of providing a semi‐quantitative treatment of the input data. Case studies will be presented using images obtained through electrochemical STM. Additionally, we propose a straightforward yet effective universal drift‐correction tool for SPM image sequences.

## 1 INTRODUCTION

Electrochemical scanning tunnelling microscopy (EC‐STM) enables the characterisation of molecules adsorbed on an electrode surface and is a potentially powerful tool for studying active sites at the nanoscopic level.^1^, ^2^ In fact, the substrate electrode can be polarised at specific potentials, creating dynamic conditions and allowing for the acquisition of corresponding images. This capability makes it possible to observe electrochemical processes, such as changes in redox states, as well as subsequent phenomena, including variations in surface coverage and molecular orientation, and chemical reactions all induced by the applied potential.^3^ The output of an EC‐STM typically consists of a series of images, obtained through the rastering motion of a tip over the sample. Namely, the tunnelling current signal is acquired along each line covered by the tip, and the tunnelling current signal is used as it is to generate constant‐height images, or, alternatively, it is fed to a feedback loop that corrects the tip vertical position (*Z* axis) so that the tunnelling current remains constant (constant‐current mode, line profiles of the tip *Z* displacements are obtained). In both cases, the scientist handles images which are indeed 2D projections of an array of line profiles. The image is formed by an image analysis software, like WSxM and Gwyddion, by reading the image *Z* range and applying a colour‐shades contrast to the overall line profiles.^4^, ^5^ Usually, the brightest shades are associated with a more positive tunnelling current value (or higher topographic features), whereas the darkest shades are linked with the lowest current values (or lowest topographic features). Despite the STM being a tool to precisely evaluate lateral dimensions of surfaces, molecules, and particles, in most cases, the *Z* scale is taken into consideration to assess a certain surface process, like a particle growth/dissolution or a catalytic reaction.^6^, ^7^, ^8^, ^9^, ^10^, ^11^

Since the analysis software displays raw STM data as 2D images with a colour contrast, the image interpretation becomes somewhat intuitive for the user, because he has visual evidence of the raw data and of the filtering and analysis operations. However, this means that image analysis is performed manually, with similar procedures and steps repeated for each image, each time applying minor adjustments to enhance the final image's appearance and interpretability. The above‐mentioned analysis software allows operations on an entire image series using only a selection of its numerous functions. Therefore, throughput is one of the main challenges in STM image analysis. Additionally, minor adjustments may unintentionally result in non‐reproducible data handling.

In this paper, the issue of STM image analysis is addressed in all aspects, ranging from basic filtering to more complex data processing operations. The approach is based on Python scripts dedicated to each specific task. In some cases where analysis operations are closely related to each other, different codes are combined into one Python notebook, to optimise the examination of the image series. Specific examples and case studies will be presented in detail to show the robustness and reliability of the developed methods.

## 2 METHODS

OpenAI in the form of ChatGPT was employed exclusively to formulate and optimise the Python scripts described in this paper and reported in Table 1. Questions and issues addressed to the AI engine concerned the sole aspect of formulating and editing Python codes and are reported in the Supporting Information. The validity of the Python codes was verified by first autonomously performing the specific tasks of each code, so that artefacts or inaccurate behaviour of the codes could be excluded.

**TABLE 1:** List of Python codes and their briefly commented functions.

Code A	Conversion of .s94 to XYZ ASCII txt files
Code B	Flattening and equalise filters
Code C	GMM clustering
Code D	Fixed thresholds segmentation
Code E	Grid expansion and drift correction (parts 1 and 2)

Input STM images were recorded either with a Wandelt‐type EC‐STM coupled with a WENKING MVS 98 voltage generator or with a RHK SPM100 STM unit coupled with a PicoStat potentiostat.^12^ In the latter case, the use of a FEMTO dlpca‐100 external amplifier should be mentioned. The electrochemical cell employed is the one developed in the Wandelt‐type EC‐STM, where the counter and the reference electrode are both platinum wires. The potential of the platinum reference electrode is calibrated against the RHE at the end of the day.

STM tips were prepared starting with a straight tungsten wire (0.25 mm in diameter) that was subjected to electrochemical etching in 2 M KOH with an AC generator imposing a square wave potential function of 100 Hz frequency and 4 V peak to peak amplitude. The obtained tips were rinsed with Milli‐Q water, and after drying in air they were coated with hot‐melt glue (160°C) to create an insulating layer on the tungsten, with the aim to prevent Faradaic current at the tip electrode. The very top of the tip will not be covered by hot glue due to its low curvature radius.

Fluka TraceSELECT Ultra (Honeywell) perchloric acid was purchased and properly diluted with Millipore Milli‐Q water (specific resistance > 18.2 MΩcm, TOC < 5 ppb) to reach a concentration of 0.1 M. Disodium phosphate (Gruessing) was purchased and properly weighed to reach a 0.2 M concentration in Milli‐Q water. pH was adjusted to 7 with small additions of 0.1 M NaOH solution.

The obtained solutions were always purged with Ar or O_2_ gas in order to evaluate the system response in the two cases. An Au(111) hat‐shaped single crystal (MaTeck) was used as substrate to be functionalised with iron octaethylporphyrin. The crystal was always subjected to flame annealing with butane flame prior to functionalisation and mounting in the electrochemical cell. N,N‐Dimethylformamide (Honeywell) was used to prepare 10^−4^ M solutions of Iron octaethylporphyrin (FeOEP). FeOEP was purchased by Sigma‐Aldrich.

Au(111) was functionalised by hanging meniscus method, immersing the surface for 60 s in the molecular solution. The crystal was then mounted in the PEEK® EC‐STM cell and electrolyte was added. Glassware was thoroughly rinsed with Milli‐Q water and Piranha solution (1 H_2_SO_4_: 1 H_2_O_2_) before introducing any other liquid.

## 3 RESULTS AND DISCUSSION

Python codes were labelled from A to E. Except for Code A, all the other Python codes were conceived to work with ASCII .txt files with XYZ arrays, both as input and output. This makes our codes highly compatible and universally usable. Code A provides an example of conversion from a .s94 data format to the requested ASCII .txt. Image analysis software always allows exporting source files as .txt files containing XYZ arrays, sometimes adding a text header before the data values to indicate the data scales.

### 3.1 Filtering – Codes A and B

Before proceeding with the extraction of significant parameters from the EC‐STM images, an initial filtering procedure must be carried out to exclude the contribution of instrumental artefacts and spurious surface defects. The two most popular image corrections performed on raw EC‐STM images are the flattening and the equalise functions, for this reason, they find a place among the few fast‐choice correction tools available in WSxM^4^ software to load and analyse a series of raw images and they are also available, with different names, in the Gwyddion^5^ software.

The flattening filter is an operation of background subtraction that is performed on a single STM image. It is a common tool provided by all the SPM software. It can be executed by exploiting different methods, spanning from the simple subtraction of a plane, which can be determined by fitting or by the user with the selection of three anchor points, to more complex polynomial fits, which are computed line by line with the possibility to discard user‐selected regions. Despite the differences among methods, their final scope is to find a good fitting function for the average surface of the analysed images and subtract it from raw data to obtain a flat image. A typical issue with STM images is the apparent inclination; namely, the contrast is dominated by a gradient of dark to bright shades. This inclination can originate from multiple factors. It could be due to a real tilting of the sample, resulting in a constant plane inclination of the acquired image, or it could be due to other factors such as *Z*‐piezoelectric scanner thermal and mechanical drift or creep.^13^, ^14^ In addition, the flattening correction gets harder when the sample morphology is characterised by a high roughness degree because of frequent terraces or nanoparticles and cannot always be effectively executed by employing a more complex fitting function. The common SPM image analysis practice heavily relies on the operator's experience, which decides the best flattening method based on the specific morphology of the image by considering the risk of losing significant information. However, under experimental conditions involving molecular monolayers at the electrified interface, the sample morphology remains unchanged throughout the experiment. This allows for the collection of a high number of images to support the investigation of the molecular monolayer's response to applied potential variations. For these reasons, automating the flattening correction is both possible and necessary.

Together with the flattening, the equalisation operation is essential, as well, to exclude outlier values in the *Z* direction. They may originate from instrumental noise or from surface defects, such as substrate terraces or molecular aggregates, which are out of interest for molecular monolayer analysis and can lead to data misinterpretation. The equalization operation allows for a selection of a smaller interval of *Z* values, thus resulting in the enhancement of the colour contrast. The software then generates a new image where the *Z* value of every point is reassigned starting from the new absolute minimum, which is set as zero, while the absolute maximum changes from image to image depending on the size of the interval selected. At the same time, the colour scale of the image is updated according to the new *Z* values interval and, for the greyscale colour map, following a linear correlation function. This task is usually performed by hand to select the correct interval of heights. However, in the case of molecular monolayers, the manual correction of images results in a time‐consuming repetitive task that can be easily automated.

In order to automate the filtering procedures of raw EC‐STM images for the subsequent statistical analysis, two python scripts were developed. A first python script (Code A) is created to convert raw STM files (.s94) into XYZ‐type ASCII files, that can be opened by the WSxM software.^4^ Those files are structured in three columns, the first, second and third columns store the *X*, *Y* and *Z* positions of the points, respectively, and every row is linked to a single image point. The interpolation of those points will generate the three‐dimensional topographic surface commonly displayed as a STM image. This Python script was adapted from the Gwyddion import module capable of reading STM files (.s94) developed by D. Nečas.^5^, ^15^

A second Python script (Code B) is developed to read the *XYZ*‐type ASCII files and perform the flattening and equalising filters by operating with an entire input file folder. The script is structured to apply the flattening correction before the equalisation one since it removes the inclination of the sample contribution. This, in some images, could alter the distribution of *Z* values resulting in a less normal‐shaped distribution, invalidating the subsequent outlier detection. In addition, since the purposes and applications of this filtering script can be multiple in terms of the characteristics of the analysed images (e.g. large‐ or small‐scale images or monolayer‐features dimensions) and further image processing and data extraction planned, Boolean operators are introduced in the first lines to: (i) enable the user selecting which kind of operations to apply, (ii) choose the percentile limits of outliers exclusion to tune the equalisation step and (iii) visualise different control plots that help to decide which is the better script configuration to employ. The flattening operation acts on an individual horizontal line in the image by iterating the calculation for every line, it fits the raw *X*,*Z* data with a parabola and subtracts the calculated fitting function from the fitted data. The equalisation filter operates on the previously flattened data: it collects the distribution of *Z* values of the entire image, then it calculates the two *Z* extrema corresponding to a specific percentile limit, which was fixed by the user. The *Z* falling beyond those limits are reassigned to the nearest *Z* extrema. Subsequently, the *Z* values are rescaled by setting the absolute minimum to zero and adjusting the remaining values accordingly, to preserve their relative differences. Eventually, corrected data are employed to save a greyscale .png image and to save an XYZ ASCII file keeping the same structure as the input ones.

### 3.2 Data analysis – Codes C and D

**FIGURE 1:**
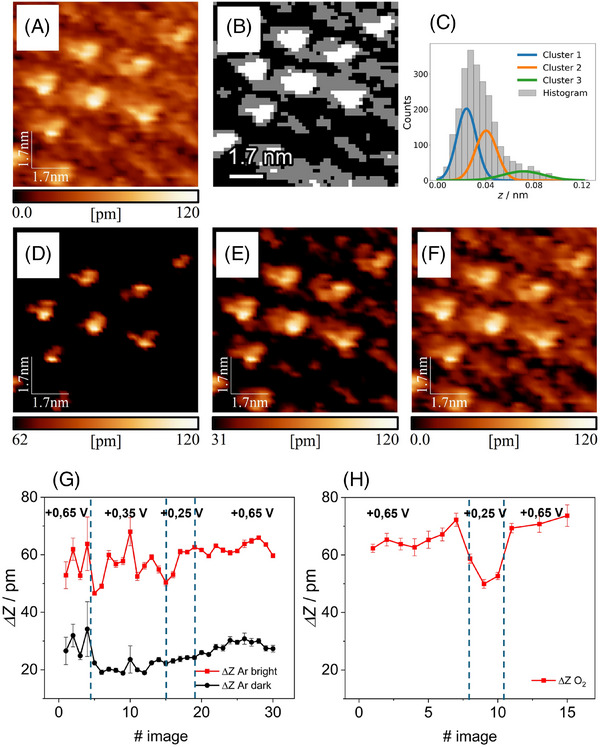
(A) EC‐STM image of FeOEP in Ar saturated 0.1 M HClO_4_. *E*_WE_ = 0.65 V vs. RHE, *I*_t_ = 5 nA, *U*_b_ = +0.150 V vs. RHE; (B) segmented representation of image ‘a’ where the white pixels belong to the cluster with higher *Z* values, the grey pixels to the middle cluster and the black ones to the lower cluster; (C) histogram plot of the *Z* values distribution of the image ‘a’ with outlined Gaussian distributions of the clusters; (D–F) images sequence obtained from ‘a’ by selecting a limited interval of the original *Z* values distribution, starting by selecting only data points from the highest *Z* values cluster and then extending to the other two cluster progressively; (G–H) graphs showing the variation of molecular heights versus the number indexing the image acquired during the potentiodynamic series measured in Ar and O_2_ saturated atmosphere respectively, cluster height differences are calculated by applying Code C.

Once the STM images have been conveniently filtered, a quantitative and statistical analysis can be reliably conducted. Our idea is to focus on the *Z* values of the images, which can be treated as numerical distributions. However, these numbers are physically meaningful, because they contain information on the tunnelling response of the surface. If the sample was subjected to a physical chemical stimulus, a variation in the *Z* distributions occurs. The key point is to consistently segment these distributions into regions that are strictly linked with molecular features composing the monolayer (e.g. the ethyl groups, the porphyrin ring, the metal centre, etc.) exploiting their peculiar height. In fact, when dealing with molecules periodically arranged on flat substrates, with a variable number of functional groups composed of different elements it appears that every molecular region possess his own tunnelling response, as documented by a number of examples.^16^, ^17^, ^18^, ^19^, ^20^ The goals here are to extract useful parameters from those regions to obtain an automated and more statistically robust determination of the topographic height comparing with the classical manual and time‐consuming topographic profile analysis method and, in addition, we want to follow the evolution of this meaningful parameters with the variation of the potential applied to the sample to quantify important physical chemical changes at the microscopic level. Therefore, we segment the distribution of *Z* values with different clustering methods to analyse the evolution of independent molecular adducts within the experimental measurement. We developed a method that employs the Gaussian Mixture Model (GMM) unsupervised clustering algorithm, from the Python library of machine learning (scikit‐learn).^21^ The GMM clustering model operates on the *Z* value distribution by grouping the data into Gaussian shape clusters. The script (Code C) was conceived with the possibility to optimise the number of clusters: the user must select a maximum number of clusters, and the algorithm will perform the calculation with different cluster numbers starting from two to the maximum cluster number. After that, the user inspects the segmented image and chooses the minimum number of clusters necessary to obtain a meaningful separation of the molecular regions of interest (e.g. the metal centre from the porphyrin ligand). After the successful identification of clusters, the mean value and the responsibility of the Gaussian component associated with every cluster are employed. The responsibility parameter calculated by the GMM is related to the probability of a random point in the distribution to be part of that cluster; therefore, it can be interpreted as the weight of the individual component determining how much each Gaussian contributes to the total data distribution. The difference between the Gaussian means reflects the average height difference among the segmented molecular regions and is used to assign the topographic height of molecules in the overlayer. Differently, the responsibility of the individual cluster accounts for the fraction of data falling in that cluster with respect to the entire sample. This parameter is exploited to calculate the percentage of data points associated with any molecular region with respect to the total image points. The Python script to perform the GMM clustering (Code C) and further analysis has the following logic. It reads the previously filtered XYZ ASCII files and performs the calculations only on the *Z* column. It computes the clustering of *Z* values with a user‐defined maximum cluster number as already explained. After that, it saves a cumulative .txt with the cluster centre positions and the responsibilities for all images, which can be further analysed with any type of data analysis software. Additionally, it generates a .png file derived from the original STM image, where data points belonging to the same cluster are represented by the same colour assigned to that cluster (Figure 1b).

**FIGURE 2:**
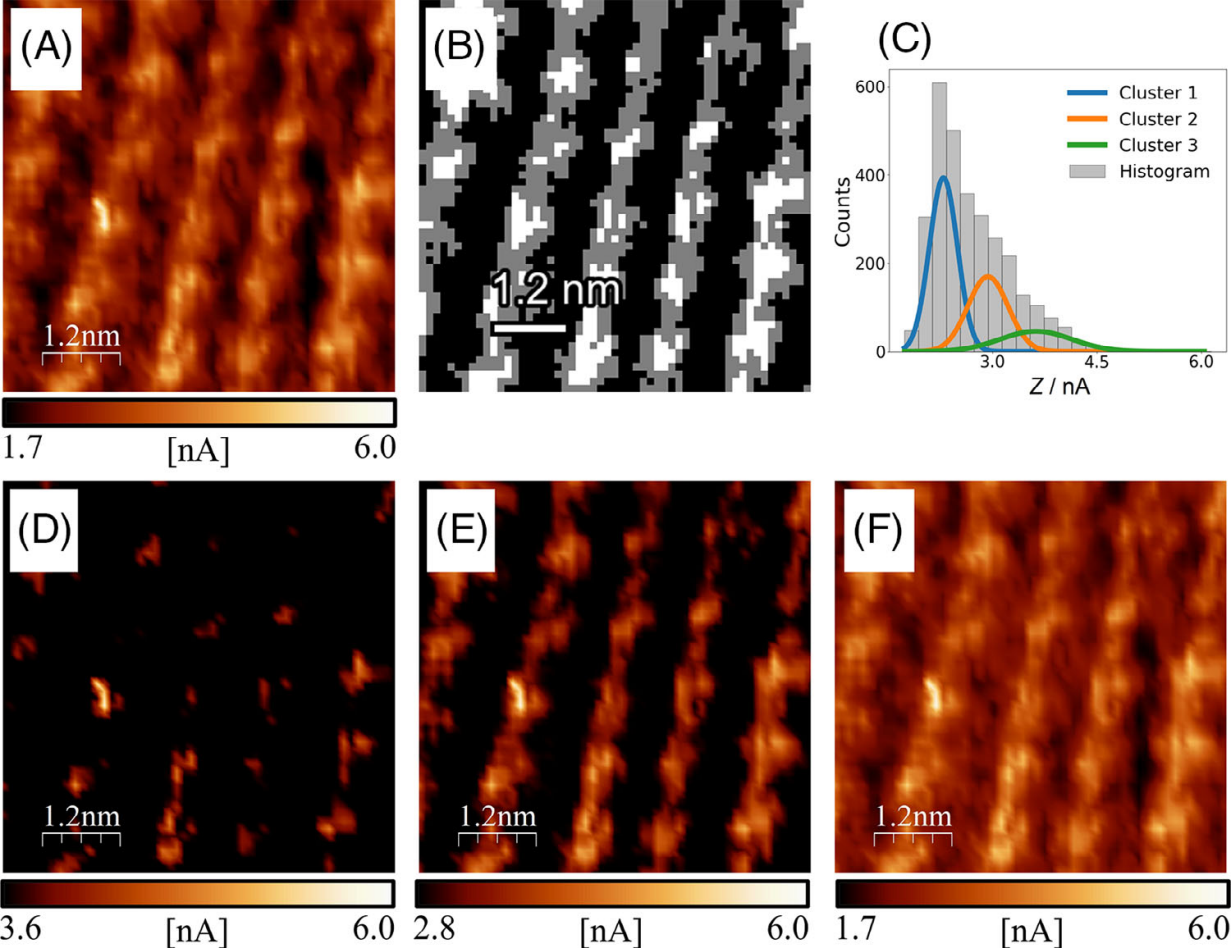
(A, A’) EC‐STM image of FeOEP and its 3 cluster segmented representation (image number 20), in O_2_ saturated 0.2 M phosphate buffer. *E*_WE_ = 0.80 V vs. RHE, *I*_t_ = 3 nA, *U*_b_ = +0.2 V vs. RHE; (B, B’) EC‐STM image of FeOEP and its 3 cluster segmented representation (image number 240), in O_2_ saturated 0.2 M phosphate buffer. *E*_WE_ = 0.50 V vs. RHE, *I*_t_ = 3 nA, *U*_b_ = +0.2 V vs. RHE; (C, D) graphs showing the variation of the pixel fractions for every cluster with respect to the total image pixels versus the number indexing the image acquired during the potentiodynamic series, calculated with Code C and Code D, respectively. The pink line represents the working electrode potential values, and it is referred to the right axis.

In addition to the GMM clustering method, a conceptually simpler method is proposed to segment EC‐STM images from the *Z* value distribution. This method is based on the direct experience of the authors, and it allows for the successful segmentation of a series of images into fixed intervals by defining constant threshold values. These are determined only on few images with a visual inspection of the operator. The main advantage of this method is the lower computation time with respect to the GMM since it doesn't need to repeat the clustering optimisation algorithm for every image. Despite not being based on a clustering algorithm that can dynamically rearrange the cluster parameters for every image to compensate for instrumental artefacts, this method is an alternative valuable candidate for mathematically handling the *Z* values, especially when dealing with large samples of images which constitute the frames of a STM video and thus are more strictly correlated between each other. Eventually this approach employing fixed intervals produces similar results to GMM clustering as shown in Figure 2d–e, which will be discussed in Section 3.4. The script (Code D) reads a sample of images starting from the first one to the number N, which is selected by the user, it calculates the maximum extension of the *Z* values distribution for every image and returns an average extension value. That extension is divided into a user‐defined number of equally spaced intervals, then it fixes the so‐obtained threshold *Z* values at the interval boundaries that are kept constant for all the images in the given series. The choice of the first *N* images to calculate the thresholds assumes that those images were acquired at the equilibrium condition, with a starting applied potential close to the open circuit potential (OCP), thus any variation of the population of data in these intervals due to the applied potential variation is compared to the initial condition. After fixing the threshold *Z* values, the script (Code D) calculates the percentage of data falling into every interval with respect to the total image data points. Code D operates similarly to Code C in the way that it reads the same type of XYZ files and, after the calculations, it saves the segmented .png image and a cumulative .txt file storing the interval parameters calculated for every image.

To validate the methods of analysis previously described, some case studies are presented where porphyrin monolayers are analysed. The successful segmentation of images is documented together with the expected variation of the height difference and the cluster data points percentage variation, in response to the external stimulus, enabling a direct comparison with the previous results obtained with the topographic profile analysis.

### 3.3 Case study 1 – FeOEP supported on Au(111) in HClO_4_

Figure 1a shows a FeOEP monolayer supported on Au(111) in Ar saturated 0.1 M HClO_4_, where dark and bright molecules can be clearly distinguished (a scheme of the molecule is reported in Figure S1). On this image, which has been already filtered with Code B, the GMM clustering performed by Code C is applied and the resulting segmentation is shown to prove the successful separation of bright molecules from dark molecules and the ethyl groups. To effectively segment this image, the total number of clusters is fixed to 3. This number was chosen through the visual inspection method (see Section 3.2) to isolate bright molecules, dark molecules and the ethyl groups by assigning them to different clusters, thereby producing the segmented image in Figure 1b. In Figure 1c, the distribution of *Z* values of the image is represented by a histogram and the Gaussian clusters are plotted as density functions normalised to the histogram area. Clusters are also shown separately in Figure 1d–f, where the images are constructed by selecting a smaller interval of *Z* values from the original distribution. Figure 1d shows only data points for the highest *Z* values cluster and then Figure 1e and f reports the addition of the other two clusters. Here it clearly emerges that the cluster indicated by the green Gaussian retains the highest *Z* and is centred on the bright centres. The intermediate cluster (orange Gaussian) is centred on the dark centres and the lower cluster (blue Gaussian) is centred on the ethyl groups between molecules. Therefore, the height of dark molecules is calculated by the difference between the middle and lowest clusters. On the other hand, bright molecule height is calculated by the difference between the highest and lowest clusters.

Subsequently, the GMM clustering was applied to a larger sample of images consecutively acquired while controlling at the same time the potential applied to the sample (*E*_we_). This is what is called a potentiodynamic series and, within that, the evolution of the monolayer topography is captured by cluster parameters variation. In particular, plotting the difference between the cluster centres produces a topographic height value, which can be directly compared with the height value resulting from the classical analysis based on the method of manually tracing topographic profiles.^8^ The plots resulting from the analysis of the two potentiodynamic series of the FeOEP monolayer supported on Au(111) in 0.1 M HClO_4_ electrolyte saturated with Ar and O_2_ gas are respectively shown in Figure 1g and h; they show the height difference between clusters versus the image number within the entire series. In addition, it's important to point out that the *E*_we_ is changed in the timescale of one image acquisition, resulting in the formation of a group of images acquired at the same potential. In the plot related to the potentiodynamic series in Ar atmosphere (Figure 1g) bright and dark molecules were successfully isolated with characteristic height of (45 ± 5) pm and (25 ± 5) pm, respectively. These are explained as Fe(III) and Fe(II) oxidation states of the porphyrin metal centre and confirmed that their height doesn't change in response to the applied potential. In the plot related to the potentiodynamic series in O_2_ atmosphere (Figure 1h) only bright molecule heights are reported since this time they are associated to oxygen‐coordinated porphyrins. In fact, their height drops when the potential reaches + 0.25 V versus RHE due to the removal of coordinated oxygen by the oxygen reduction reaction (ORR) taking place at the electrode.

### 3.4 Case study 2 – FeOEP supported on Au(111) in 0.2 M phosphate buffer

**FIGURE 3:**
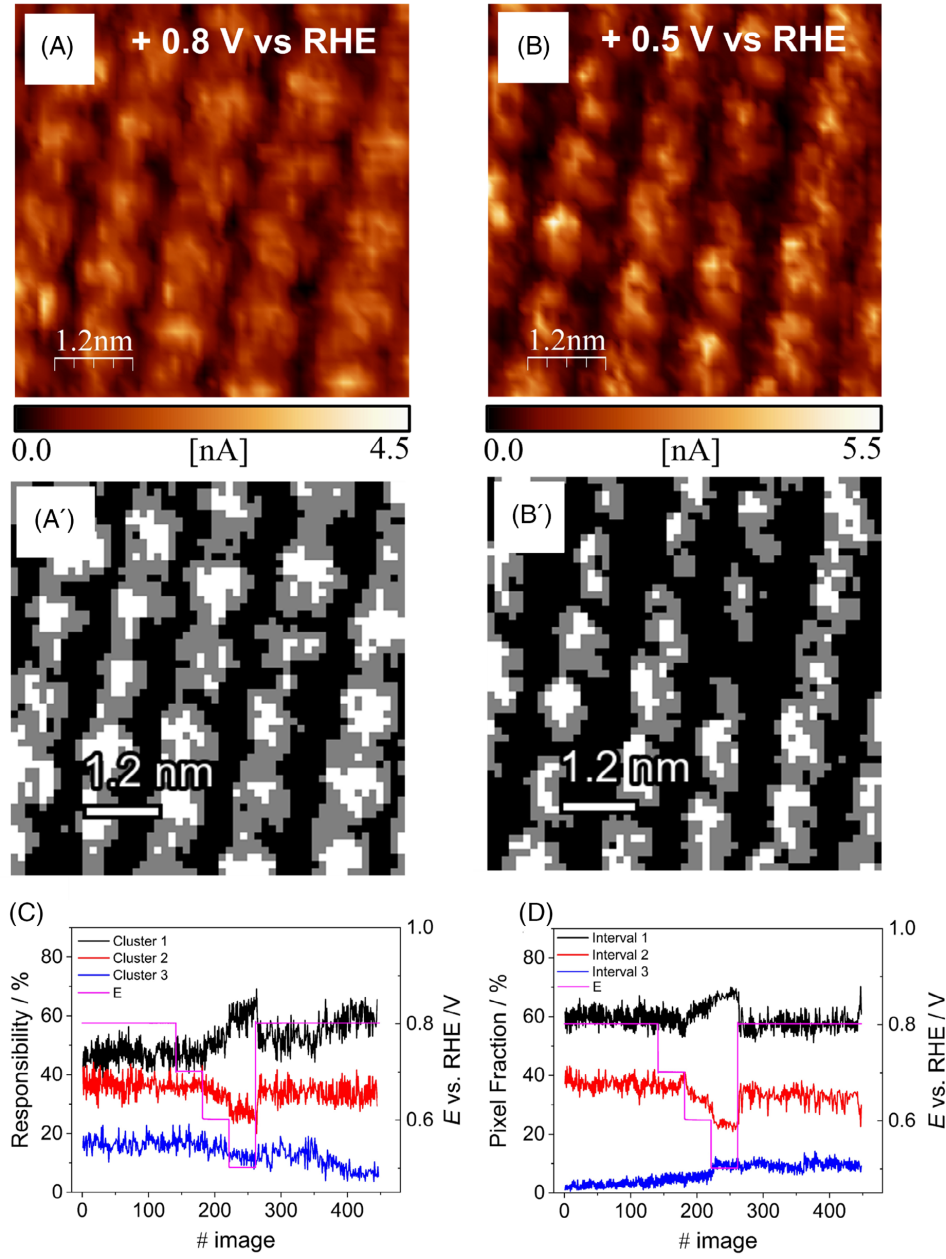
(A) EC‐STM image (image number 100) of FeOEP in O_2_ saturated 0.2 M phosphate buffer. *E*_WE_ = 0.80 V vs. RHE, *I*_t_ = 3 nA, *U*_b_ = +0.2 V vs. RHE; (B) segmented representation of image ‘a’ produced by Code C, where the white pixels belong to Cluster 3, the grey pixels to Cluster 2 and the black pixels to Cluster 1; (C) histogram plot of the *Z* values distribution of the image ‘a’ with outlined Gaussian distributions of the clusters; (D–F) images sequence obtained from ‘a’ by selecting a limited interval of the original *Z* values distribution, starting by selecting only data points from the highest *Z* values cluster and then extending to the other two cluster progressively.

The same FeOEP layer was tested under substantially different experimental conditions. A neutral solution, namely pH = 7, based on phosphate buffer was employed as electrolytic solution. Then, STM imaging was performed with constant height mode, implying that the measured *Z* value was the punctual tunnelling current. Moreover, the number of computed images was higher since they constitute the individual frames of a STM video acquired at 8 frames per second. Nevertheless, the GMM clustering method (Code C) adopted in Case Study 1 was also successful in segmenting this sample of images. In addition, the fixed threshold segmentation method (Code D) is employed and compared to the GMM leading to comparable results. Figure 3a displays the FeOEP adlayer with the full *Z* range, enabling to immediately notice regions with significantly bright contrast, and rather dark contrast, as well. A satisfactory *Z* segmentation of this image series was achieved with Code C by employing 3 clusters. In Figure 3b and c, we can respectively see the resulting segmented STM image and the *Z* distribution fitted with the three Gaussian clusters. In Figure 3d–f, it is evidenced the cluster assignation to molecular features by showing a STM image with a limited *Z* values interval that initially is centred on the third cluster (Figure 3d) and then it extends to the second (Figure 3e) and the first one (Figure 3f) progressively. By looking at this image sequence it can be clearly seen that the first cluster gathers the bright centres appearing on some molecules, the second cluster accounts for the ligand region of every molecule and the third cluster includes the ethyl group between molecules.

Code C and Code D are tested on the same image series which consists of 450 images acquired while a chronoamperometric experiment was performed, thus the potential applied to the sample was varied instantaneously from a value near the OCP to a potential triggering the ORR and kept constant to acquire multiple STM images in both conditions. The working electrode potential variations performed during the chronoamperometric experiment are visible as a pink line in Figure 2c and d. By a visual comparison of two images taken before and after the potential variation (Figure 2a and b), it can be seen an overall darkening of the image due to the lowering of the tunnelling signal coming from the molecular ligand and this is captured by the GMM segmentation, showed in Figure 2a’ and b’, which assigns more pixels to the lower cluster and less to the middle one. The graphs following the evolution of the different pixel population calculated with GMM and threshold method throughout the entire image series are reported in Figure 2c and d. With both methods, we can appreciate the same response of the image pixel population to the applied working electrode potential (pink line). The trend of this variation is consistent with the behaviour already described in the visual comparison commented before, with an increment of dark pixels (cluster 1) and a decrease of grey ones (cluster 2). Therefore, here we are observing a decreasing of the tunnelling signal coming from the molecular monolayer when a sufficiently reducing potential is applied to activate the ORR, this could be interpreted as a reduction in the concentration of illuminated molecules caused by the removal of coordinated oxygen.

### 3.5 Drift‐correction – Code E

In this paper, we also present a very simple python‐based tool developed to perform an offline (i.e. after the measurement) drift correction affecting a series of images. This method was designed to correct translational drift affecting the entire image, rather than distortions caused by temperature variations and/or piezoelectric creep. For this latter task, a variety of scripts, software, and methods already exist. Some authors employ the known sublattice dimensions as an internal standard to perform the drift correction.^13^, ^22^ Others calculate the drift correction by comparing two images taken on the same object but at different scanning times or orientations, for example, comparing the forward and backward or up and down, and looking at the correlation matrix.^23^, ^24^, ^25^ For our scope the first strategy is not suitable since we usually have a molecular overlayer that hide the underneath single crystal substrate, neither the second strategy is useful since the drift we want to correct is not the fast one responsible for image distortions, rather the slower one that make loose the initial position when a long series of images is taken in an identical location and it exhibits a strong nonlinear behaviour. Therefore, we choose to develop a manual method of drift correction that exploits recognisable features to be used as an anchor marker by the user to manually correct the *x*, *y* position of a selected sample of images over the entire image series. It must be mentioned that a recently developed drift correction software employs a combination of cross‐correlation calculation and anchor marker identification to perform an offline correction of fast and slow drift without internal standard calibration.^26^ However, the advantage of our method consists in the manual tracking of markers that allow for stabilising atomic and/or molecular features, so that they retain their (*x*, *y*) position with high precision throughout the entire image sequence consisting of hundreds of images.

Eventually, we must recall another recent method that was demonstrated for the correction of slow drift over a long series of images. This method is based on the cross‐correlation between the FFT of selected images along the series. The idea is to exploit the periodicity of the images by moving to the reciprocal space to align their relative position in the real space.^27^ However, if that periodicity varies in time and/or space, then artefacts or inefficient correction are obtained. This brings us to discuss one typical example of EC‐STM measurements, that is the imaging of periodic and ordered arrays of molecules physisorbed on a flat substrate like HOPG or Au(111). In this case, the imaged features are periodic, nevertheless, modifications in the array may occur, especially if the sample is subjected to electrochemical potential control to trigger electrochemical or electrocatalytic processes. In those cases, changes in the molecular adlayer, loss of molecules, and adsorption/desorption phenomena are very frequent, and a correlation based on FFT alignment would be misleading and ineffective. However, at the same time, a discrete number of objects remain intact and could be potentially used to align consecutive images. For this reason, we developed a script (Code E) to manually index a specific feature of an STM picture that can be visually identified throughout the entire image sequence.

Before proceeding with the drift correction, a preliminary operation of grid expansion must be accomplished. The code simply reads each *XYZ* ASCII file, and expands the image by adding 0 values around the original real data. The result is the original image enclosed with a black (empty) frame. This operation allows for easy processing of the images for the final drift correction. A rigid translation of images will be applied, and having a blank outer region facilitates this operation.

The Python script (Code E) is divided into two parts. Part 1 is structured so that a desired number of images of an entire series are interactively displayed on a web‐based interactive Python environment like JupyterLab.^28^ The number of displayed figures can be conveniently adjusted before running the script depending on the number of total images and on the expected/observed drift. An interactive square with a sub‐square pattern appears together with the visualised images and can be dragged with the mouse to place it on a visually recognisable feature of each STM picture. The dimensions of the square can be adjusted before running the script to match the size of the desired feature to monitor. The absolute position of the centre of the square of each image is recorded immediately after releasing the mouse, and the corresponding (*x*, *y*) coordinates are listed in the form of tuples (file name, *x* centre, *y* centre) by launching a subsequent small code.

At this point, Part 2 of Code E comes into play. This building block receives the (*x*, *y*) coordinates obtained in the previous section and uses the first (*x*, *y*) point as a reference. Subsequent points are re‐computed to estimate the deviation from that point. An interpolation operation is executed to extend the deviation calculation to all the image series. Additionally, the interpolated points are fitted either with a cubic spline or with polynomial functions. The fitting functions can be selected with dedicated Boolean flags. A plot of the fitted (*x*, *y*) coordinates is displayed in Jupyter Lab. The code then moves each individual picture from its original position according to the new positions determined through the fitting. This also guarantees that the adjusted frames remain at the centre of the outer black region, showing a trace of the original position of the picture before correction. This is useful for performing a resize with any STM‐analysis dedicated software, like WSxM or Gwyddion.^4^, ^5^

## 4 CONCLUSION

In this paper, we systematically explored the topic of STM image analysis, and we proposed various Python‐based codes to handle STM images and execute numerical operations to extract valuable information. Code A was conceived as a preliminary tool to convert .s94 raw data files originated by an STM instrument into ASCII .txt files with XYZ arrays. Code B enables filtering operations of raw images, in particular the background subtraction and the *Z* range rescaling to exclude outlier data points. Code C applies the Gaussian Mixture Model to the *Z* value distribution by grouping the data into Gaussian shape clusters, returning the cluster centres positions and their responsibility parameters in a cumulative .txt file and segmented images as control plots. Code D has a task similar to Code C, but it divides the *Z* range extension into a user‐defined number of equally spaced intervals, then it fixes the so obtained threshold *Z* values at the intervals boundaries that are kept constant for all the images in the given series. This allows a faster sorting of the *Z* data points, without running a clustering algorithm that employs a higher computation time; this method is especially suited for the analysis of images constituting individual frames of an STM video which are gathered in large numbers and highly intercorrelated between each other. Two case studies regarding the investigation of FeOEP molecular monolayer covering an Au(111) single crystal and immersed in different supporting electrolytes (i.e. 0.1 M HClO_4_ or 0.2 M Phosphate buffer) were presented. Here, the newly developed Python codes were tested and validated. In particular, the application of Code C and Code D demonstrated a successful segmentation of EC‐STM images in both case studies. Furthermore, the codes allow for processing a larger number of images with respect to manual topographic analysis method. As a result, the analysis of image series from potentiodynamic experiments captures the electron density variation taking place on the average molecule in the monolayer, when the applied potential is changed from a value near the OCP to a value near the peak of ORR, due to the removal of adsorbed oxygen, leading to the same interpretation of the redox process provided by the manual method. Finally, Code E brings forth a method of drift correction, by employing an interactive manual detection of a recursive feature along the image series to stabilise it along the image series. This paper thus represents a first intermediate step towards a fully, or at least widely, automatised analysis tool for STM data.

## AUTHOR CONTRIBUTIONS

F.C. contributed to the conception and design of the work and code development; A.F. and S.R. contributed to the data acquisition; A.F. and F.C. data analysis and interpretation. All authors reviewed and approved the submitted manuscript.

## CONFLICT OF INTEREST STATEMENT

The authors declare no conflicts of interests.

## CODE AVAILABILITY

Python codes are available as supporting information file; all original STM data are available upon requests at the Research Data Unipd repository (link: https://researchdata.cab.unipd.it/id/eprint/1489).

## Supporting information



Supporting Information

## Data Availability

The data that support the findings of this study are openly available in Research Data Unipd at https://researchdata.cab.unipd.it/id/eprint/1489.
