# Supplementary material for: Simple Python‐based methods for analysis and drift‐correction of STM images
Source: J Microsc. 2025 May 14;302(1):39–49. doi: 10.1111/jmi.13426 (PMC13045769; doi:10.1111/jmi.13426)
Supplement: Supplementary file 1 — Supporting Information [file JMI-302-39-s001.docx]

**Supporting Information**

**Simple Python-based methods for analysis and drift-correction of STM images**

*Francesco Cazzadori,^a^ Alessandro Facchin, ^b,^* Silvio Reginato,^a^ Christian Durante^a,^**

^a^University of Padova, Department of Chemical Sciences, via Marzolo, 1 - 35131 Padova, Italy;

^b^TUM School of Natural Sciences, Department of Chemistry, Chair of Physical Chemistry

Lichtenbergstr. 4, 85748 Garching, Germany

*Email: christian.durante@unipd.it*

S1 Supporting Information 2

S2 Supporting Figures 5

S3 Supporting Table 7

S1 **Supporting Information**

The Python codes described in the article (codes A-E) are available for the download on the institutional research data archive “Research Data Unipd” (link: <https://researchdata.cab.unipd.it/id/eprint/1489>).

The questions addressed to the AI engine are summarized here for each developed code. They should not be regarded as a transcription of the actual conversations with ChatGPT, but rather as a synthesis of the key questions that guided the final design of each script.

*Code A*

The source code of Gwyddion import module for .s94 file is found online in a public repository at the following link:

<https://browse.dgit.debian.org/gwyddion.git/plain/modules/file/s94file.c?id=6773723bcf9a8b1b803eb6a5bc93e5c7d688b325>

This code was copied and pasted into the AI engine chat, and the following request was formulated: “This is a script written in C language, translate it into python language”

*Code B*

“Write a Python script to perform data analysis on a .txt file, that can be also visualized as a 2d image, where the first column stores the x position for every pixel, the second column stores the y position for every pixel. The third column stores the z position for every pixel.

Define an input folder and an output folder. Inside the output folder, also create a "ControlPlots" subfolder for optional control plots.

User settings and parameters:

0) Add Boolean variables allowing the user to turn on/off:

a) Parabolic flattening (perform_flattening)

b) Percentile clipping for Z values (percentile_clipping)

c) Saving a control plot of the flattening on the 128° row (control_plot_flatten)

d) Saving a control plot of the Z value histogram (control_plot_fitted_histogram)

1) Define clipping_percentile (e.g., 99) for clipping Z values.

Script acting on each .txt file in the input folder starting a for loop:

1) Read the individual file by discarding the header lines, which are the first three lines, and the three columns separated by tab. The first, second and third column contains the x, y and z values of the image.

2) Calculate the pixel to nanometer ratio by assuming evenly spaced data and use this information to set the scale bar length equal to the 20 % of the total x range.

3) If perform_flattening is true. Perform a simple-flatten processing step to the image: fit every horizontal line in the image with a parabola. This parabolic fit must consider x values of the pixels on the x axis and the z values of the pixels on the y axis (horizontal lines are the ones obtained by varying x pixel values while keeping the y value constant). For every line subtract its own fit. Then, if control_plot_flatten is true, plot the graph of the 128° line and its calculated fit as a control plot, saving as a .png image in the control_output_folder.

4) If percentile_clipping is true. Operate only on the Z values and perform a percentile clipping step to remove outliers. Clip the data at a specified bound which depends on the percentile that can be selected by the user, as an example the 95 %. Then rescale the z values by setting the absolute minimum to zero and rescaling the other one keeping the same differences between points. If control_plot_fitted_histogram is true, save a histogram of the z values prior to the clipping operation and overlay the bound used for clipping.

5) Save the processed data in the output folder as follows:

a) The processed x,y,z data in a new .txt file with the following header: WSxM file copyright UAM (in the fist line), WSxM ASCII XYZ file (in the second line), X[nm] Y[nm] Z[nm] (in the third line).

b) The processed x,y,z data reshaped to form a 2d grid and to save a .png image where x,y are the pixel positions and z is the intensity of every pixel expressed in a linear grey color scale where the absolute minimum value of z in the whole file is associated to the black color, the absolute maximum value of z in the whole file is associated to the white color. Add a scale bar on the bottom left of the image with horizontal length equal to the 20% of the horizontal image width. The width of the scale bar must be equal to 0.03 of the scale bar length. The scale bar must have vertical ticks at the borders. The length of the scale bar must be written on top of the bar with nm as unit measure.”

*Code C*

The first part of Code B that reads the .txt data was copied in the chat and, after that, the request was formulated as following: “Modify this python script to perform a gaussian mixture model segmentation on the Z values with a number of clusters that start with N = 2 and repeat the calculation adding one cluster per cycle until a user-defined number of cluster = N. For every loop, label the cluster starting from the one that has the lowest cluster center (cluster 1) and moving, in increasing order, towards the one that has the highest cluster center (cluster N). For every loop, save a segmented image where all the pixel pertaining to the same cluster have the same color; use a gray scale palette and assign the black color to cluster 1 and the white color to cluster N. Build also a cumulative .txt file storing for every processed .txt in the run the cluster centers and the cluster responsibilities.”

*Code D*

The first part of Code B that reads the .txt data was copied in the chat and, after that, the request was formulated as following: “Modify this python script to preprocess a user-defined number N of .txt files to gather the Z values of all files in one single array, then exclude a user-defined percentile from this array. Then, divide the average Z range into a user-defined number n of intervals by fixing equally spaced threshold values. Analyze all the .txt file stored in the input folder by keeping the previously calculated fixed thresholds values, compute the interval percentages by counting how many image pixels pertain to every interval with respect to the total pixels in the image. Save a cumulative plot of the percentage associated with every interval for every .txt file analyzed in the input folder. Save a segmented .png image for every .txt file where all the pixel pertaining to the same interval have the same color; use a gray scale palette and assign the black color to interval 1 and the white color to interval n.

*Code E*

1) Grid expansion and image recalibration

“Write a Python code that expands input files with a black ribbon. The input files are STM image structured as a xyz data with .txt extension. Note that the input files have a header like

“WSxM file copyright UAM

WSxM ASCII XYZ file

X[nm] Y[nm] Z[nm]”

with Z that could be also [nA]. The header should be maintained in the output files as well.

I want the output images to have a doubled size, where the black ribbon around the original data is given by zero values of Z. The output files must be recalibrated, so for every xyz coordinate multiply x and y values by 2, then write the modified data in new output files.”

2) Drift correction

a) Part 1

“Write a Python code that opens STM xyz files with .txt extension, skipping the non-numeric headers. Then the code will display a sample of images (one every N images, where N is a modifiable parameter) in different interactive windows. In every window, a green square pattern forming a cross must be placed with the mouse on the desired target present on each picture. For every image the coordinates of the spot have to be stored and reported in a table-like format with 6 significant digits.”

b) Part 2

“Write a Python code that performs a Spline or polynomial fit of user-defined coordinates inserted in a table-like format, where for every .txt file corresponds a xy pair. Two different fit must be performed, one for the x values and one for the y values. I also want you to plot the results of the interpolation. Using the first frame as reference, compute the target position from the fit. Then compute the shift as the difference between the reference position and the target position. This shift must be added only to the non-zero Z values. Before saving sany output consider the possibility to expand the outer black region if the xy coordinates of the non-zero Z values exceed the ones of the frame.”

**S2 Supporting Figures**

**
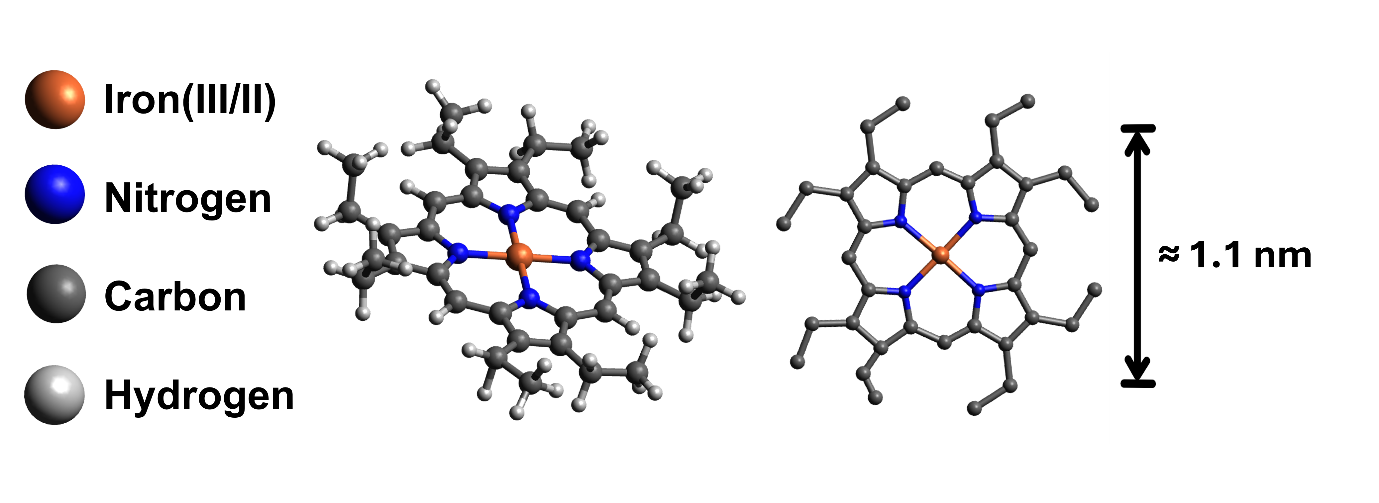
**

**Figure S1**. Iron Octaethylporphyrin (FeOEP) scheme with atom color legend on the left

To perform the drift correction, 6 out of 126 input images (1 image every 25) were chosen as a representative sample to be employed in the manual drift correction procedure. Part 1 of code E consists in centering the marker represented by the center of the green cross on a specific feature. The cross is interactively dragged with the mouse onto the center of one molecule, which can be identified in all the 6 images (Figure S2). Part 1 then displays the corresponding (x,y) coordinates, which are shown in table ST1 and plotted in Figure S3a to visualize the entity of the drift, here the image number on the X axis is visualized as measurement time due to the known acquisition frequency of 8 frames per second. In Figures S4a and S4b are compared the image #1 and #150 of the series prior and after the application of the drift correction showing a satisfactory compensation of a 1.4 nm coordinates shift. The correction is carried out by the second part of Code E, which interpolates the coordinates positions shown in table ST1 with a polynomial function of the 3^rd^ order and calculates the entity of the rigid translation that must be applied to every image of the series.


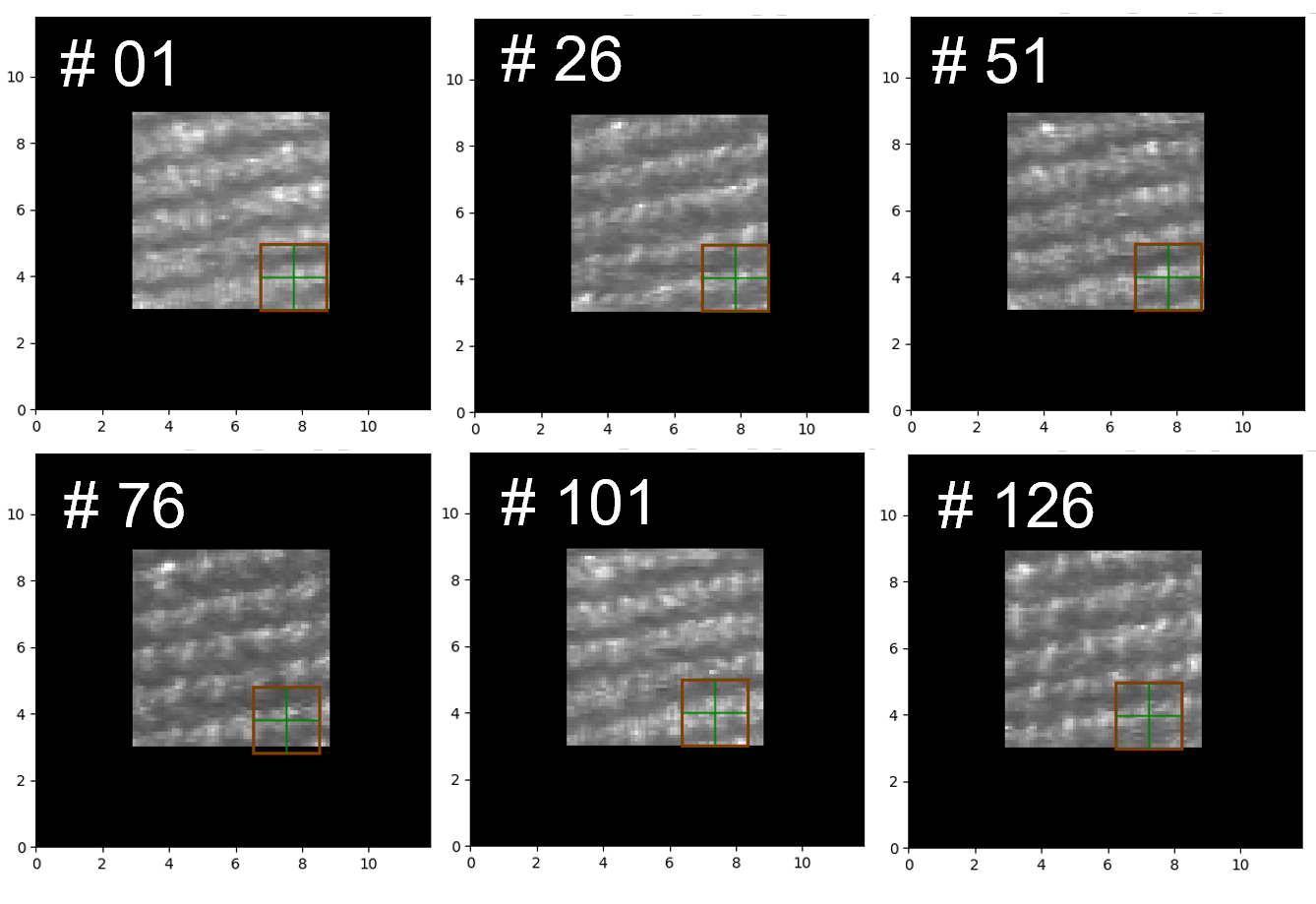


**Figure S2**. Sequential images: the green cross was dragged on the identical feature alongside the image series.


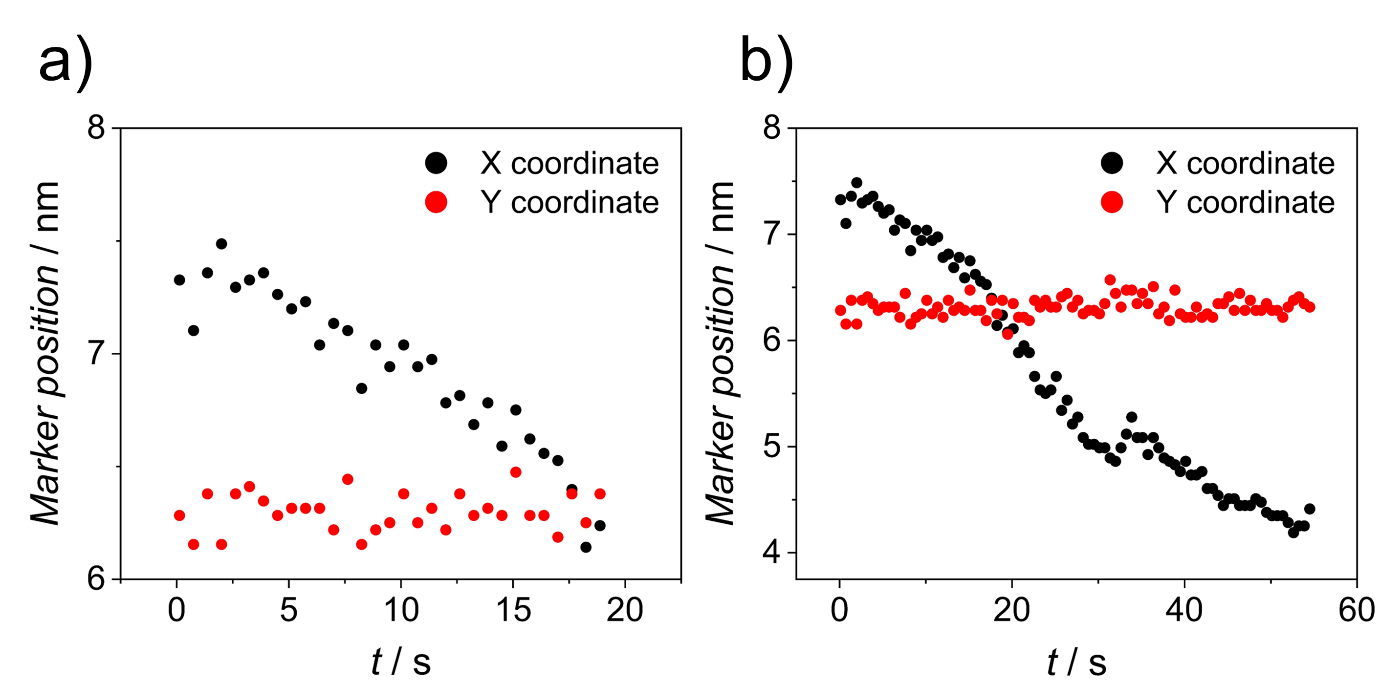


**Figure S3**. Plot of the x and y drift expressed as the marker position obtained by tracking the center of a single molecule for a one-minute acquisition of 8 fps: a) first 150 frames of the image series; b) complete series.


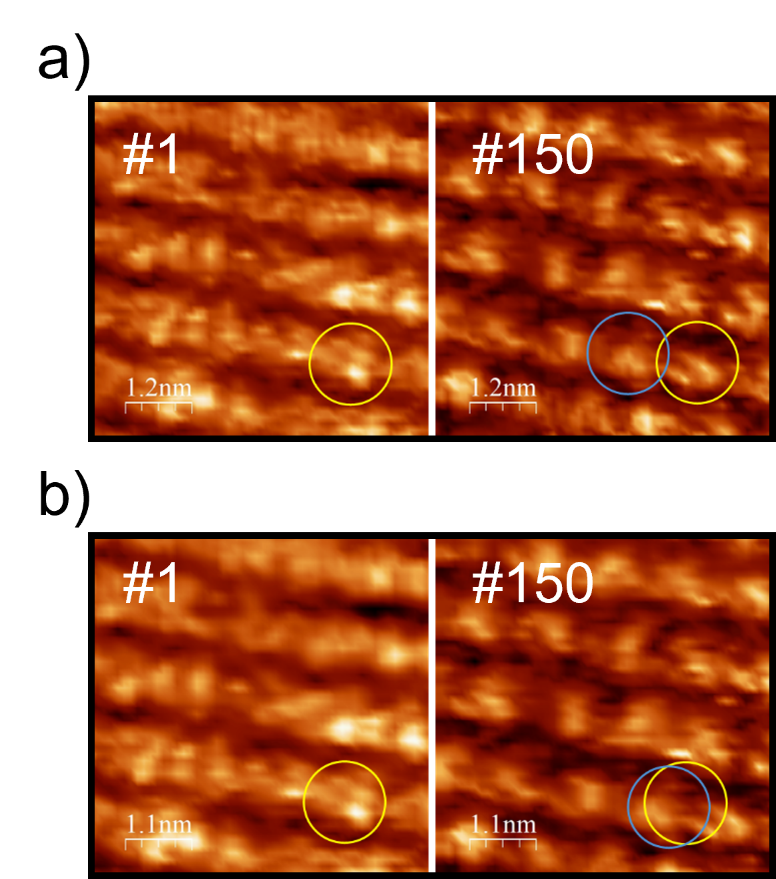


**Figure S4**. Comparison of the image number 1 and 150 of the series prior the drift correction, in box a), and after the drift correction, in box b). The yellow circle highlights a single FeOEP molecule in the image on the left; while, in the image on the right, the same molecule is highlighted with a blue circle and the yellow circle is kept fixed in position to facilitate the visualization of the shift.

**S3 Supporting Tables**

Table ST1. Coordinates corresponding to the center of the green crosses of Figure S2.

| Clicked Points | Coordinates | |
| --- | --- | --- |
| File Name | X (nm) | Y (nm) |
| f0001 | 7.743106 | 3.977228 |
| f0026 | 7.839174 | 4.041274 |
| f0051 | 7.743106 | 4.009251 |
| f0076 | 7.518947 | 3.817114 |
| f0101 | 7.358833 | 4.009251 |
| f0126 | 7.230742 | 3.977228 |
